# HIV-associated large-vessel vasculopathy: a review of the current and emerging clinicopathological spectrum in vascular surgical practice

**DOI:** 10.5830/CVJA-2015-017

**Published:** 2015

**Authors:** Balasoobramanien Pillay, Pratistadevi K Ramdial, Datshana P Naidoo

**Affiliations:** Department of Vascular/Endovascular Surgery, Nelson R Mandela School of Medicine, Durban, South Africa; Department of Anatomical Pathology, School of Laboratory Medicine and Medical Sciences, University of KwaZulu-Natal and National Health Laboratory Service, Durban, South Africa; Department of Cardiology, University of KwaZulu-Natal, Durban, South Africa

**Keywords:** human immunodeficiency virus, vasculopathy, aneurysms, occlusive disease, atherosclerosis, vascular surgery

## Abstract

An established relationship exists between human immunodeficiency virus (HIV) and the vascular system, which is characterised by clinical expressions of aneurysmal and occlusive disease that emanate from a common pathological process. The exact pathogenesis is currently unknown; attempts to implicate opportunistic pathogens have been futile. Theories converge on leucocytoclastic vasculitis with the vaso vasora as the vasculopathic epicentre. It is thought that the virus itself or viral proteins trigger the release of inflammatory mediators that cause endothelial dysfunction and smooth muscle proliferation leading to vascular injury and thrombosis. The beneficial effects of highly active anti-retroviral therapy alter the natural history of the disease profile and promote longevity but are negated by cardiovascular complications. Atherosclerosis is an emerging challenge. Presently patients are managed by standard surgical protocols because of non-existent universal surgical interventional guidelines. Clinical response to treatment is variable and often compounded by complications of graft occlusion, sepsis and poor wound healing. The clinical, imaging and pathological observations position HIV-associated large-vessel vasculopathy as a unique entity. This review highlights the spectrum of HIV-associated large-vessel aneurysmal, occlusive and atherosclerotic disease in vascular surgical practice.

## Abstract

Since the first description of human immunodeficiency virus (HIV) disease more than three decades ago,^1^ HIV infection has become a global phenomenon, afflicting approximately 40 million people worldwide.^2^ Over 70% of infected individuals reside in sub-Saharan Africa.^2^

HIV is implicated in a multisystem disease process and the cardiovascular system is not spared. Many infected patients are in the advanced stages of disease and present with vascular complications.^3^,^4^ The unique vascular manifestations of HIV-related disease, documented as early as 1987 in children,^5^ may present with a diverse spectrum of aneurysms, occlusive disease, spontaneous arteriovenous fistulae and dissections. In addition, despite the heightened recognition of the spectrum of HIV and other HIV-associated infective vasculitic and vasculopathic reactions,^6^-^10^ the exact pathogenetic mechanisms and reasons underpinning the varied manifestations of the HIV-associated vascular pathology remain enigmatic.

This review discusses the spectrum of HIV-associated large-vessel disease in vascular surgical practice, including the current understanding of pathogenetic mechanisms, the clinicopathological profile of aneurymal and occlusive disease, and the impact of highly active anti-retroviral therapy (HAART) in the development of atherosclerosis in this patient population.

## Pathogenesis of HIV-associated large-vessel vasculopathy

The pathogenesis of HIV-associated large vessel vasculopathy involves intricate, dynamic interactions between viral-induced inflammatory responses, vascular smooth muscle changes, endothelial alterations and circulating blood factors that result in pathological vessel wall alterations and symptomatic clinical disease (Fig. 1A, B).^11^,^12^

**Fig. 1A.**
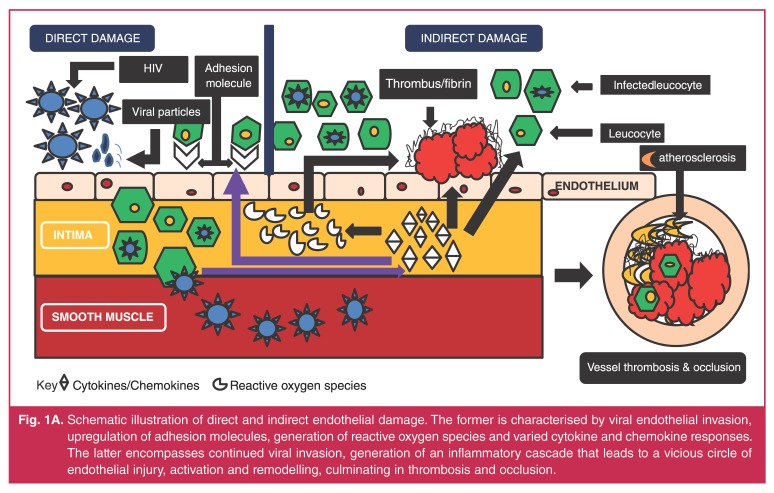
Schematic illustration of direct and indirect endothelial damage. The former is characterised by viral endothelial invasion, upregulation of adhesion molecules, generation of reactive oxygen species and varied cytokine and chemokine responses. The latter encompasses continued viral invasion, generation of an inflammatory cascade that leads to a vicious circle of endothelial injury, activation and remodelling, culminating in thrombosis and occlusion.

**Fig. 1B.**
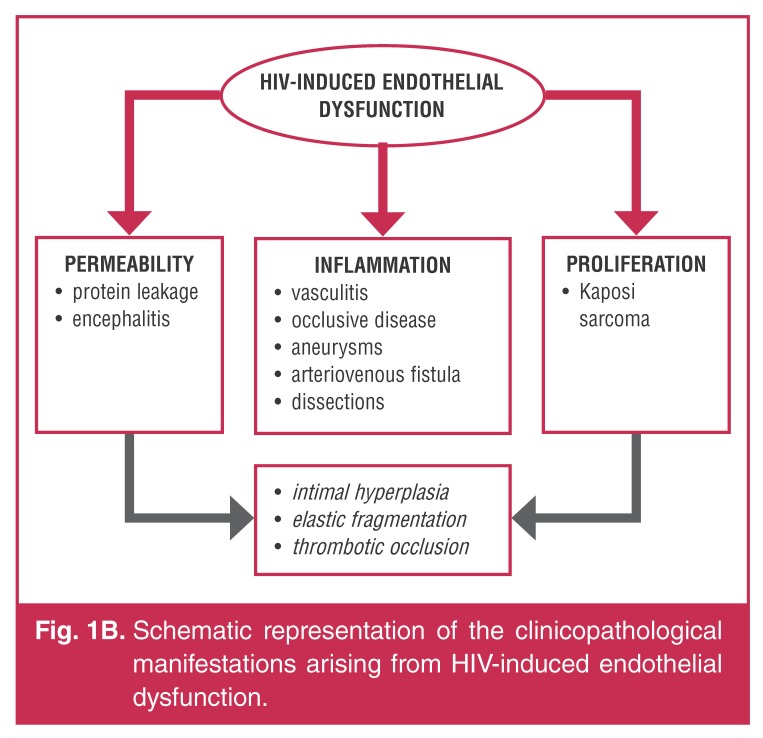
Schematic representation of the clinicopathological manifestations arising from HIV-induced endothelial dysfunction.

## Inflammatory alterations

The hallmark pathological feature common to aneurysmal and occlusive disease is an HIV-associated vasculitic process, the exact mechanism of which is poorly understood (Fig. 1A, B).^13^,^14^ HIV-related endothelial dysfunction, incorporating a complex interplay between cytokines and inflammatory components, has been proposed.^14^ The theoretical basis of this includes continuing viral infection and associated viral protein toxicity leading to vascular wall injury, an increase in viral load associated with the release of interleukin 1 (IL1), interleukin 6 (IL6), interleukin 8 (IL8) and tumour necrosis factor-α (TNF-α), in conjunction with immune activation and immune reconstitution as a result of HAART.^14^

This has been observed by Nieuwhof *et al.*^15^ who postulated increased T-cell numbers associated with elevated CD25-positive receptors in the setting of cerebral vasculitis. This theory is supported by the response to steroids and daclizumab, a human immunoglobulin G-K recombinant antibody that binds to CD25.^15^ It is thought that the HIV-transactivator of transcription protein, *tat*, triggers inflammatory pathways that result in the production of cytokines and adhesion molecules. A viral envelope glycoprotein component (gp120) is a catalyst that stimulates production of pro-inflammatory mediators, which target endothelial cells.^14^,^16^,^17^ Evidence from studies on flow-mediated vasodilatation demonstrates that HIV-associated endothelial dysfunction is catalysed by these cytokines and the inflammatory process.^13^,^14^,^17^

## Role of smooth muscle cells

Smooth muscle cells (SMCs), the major cellular component of the arterial muscularis media, have proliferative and migratory potential. Key surface receptors, CD4, CCR5 and CXCR4, render SMCs an ideal infective target that facilitates entry of the HIV-1 viral components.^18^ Viral invasion results in thinning of the medial layer and sub-intimal aggregation of SMCs. Entry of the viral envelope proteins may activate tissue factor 2, which is a potent stimulant for the coagulation cascade.

While the SMCs seems to play a central role in arterial wall pathophysiology, as evidenced by in vivo and in vitro SMC studies,^19^ this aspect of smooth muscle involvement has not been studied in depth. More recently Gutierrez *et al.*,^20^ in their appraisal of intracranial vessels from autopsy specimens in a pilot study of 15 arterial wall samples from five patients, found that there was thinning of the arterial media. Although this observation may be indicative of a significant pre-clinical stage towards HIV-associated vasculopathy with resultant vessel wall weakening, it has to be interpreted with caution in view of study limitations in terms of numbers, lack of information on HAART and duration of HIV infection.

The entry of the virus into the cell triggers release of tissue factor 2, which induces thrombosis and chemokine c-c motif ligand (CCL-2) production. This is instrumental in promoting atherogenesis. These observations may support a role for direct viral invasion, and may contribute to knowledge on the thrombotic complications and coronary events experienced by HIV-infected patients.

## ‘Molecular mimicry’

Tilson^21^ studied an HIV-related carotid aneurysm and explains molecular mimicry whereby HIV viral proteins share antigens in the wall of the vasculopathic process. The load-bearing matrix of the arterial wall is composed of an artery-specific antigenic protein (ASAP), matrix cell adhesion molecule-1 (Mat-CAM-1). It is theorised that the virus and its toxic by-products share ligands that are characterised by DNA sequence similarities between the ASAP and viral envelope glycoprotein, gp41 and gp120.^21^ This may potentially result in autoimmune-mediated cell damage during infection. However, no similarities were found between Mat-CAM-1 and HIV envelope glycoproteins in this study. Therefore an alternate explanation proposed is that of direct viral invasion of the aortic fibroblasts at the level of the adventitia.^21^

## Thrombophilic screening

While the pathogenesis of occlusive disease is presently unclear, thrombophilic screens have been sporadically performed with regard to protein S, protein C and antithrombin III. Mulaudzi *et al*.,^22^ however, found negative thrombophilic screens in 10 patients with primary arterial thrombosis in the acute setting. Chronic infection in HIV-infected patients results in endothelial injury and associated dysfunction. This sequence of events culminates in atherosclerosis and thrombosis.

## Experimental models

Animal models^23^ have been employed to simulate the arterial wall pathology in order to improve insight into the underlying mechanisms of HIV-associated vasculopathy. Studies conducted in transgenic mice infected by the HIV-1 provirus have demonstrated an adventitial mixed inflammatory cell infiltrate, medial hypocellularity and intimal hyperplasia following smooth muscle migration, with sparing of the endothelial cells. The intimal thickening produces intraluminal narrowing of some vessels causing distal tissue ischaemia.

In addition, viral components have been observed in SMCs, which in some instances have proliferated in the absence of inflammation. This model partially explains the findings in human arterial wall samples, with the key feature being endothelial dysfunction. Although this model highlights the conceptual principles of viral invasion, extrapolation of the pathophysiological findings of arterial wall studies to the human scenario remains challenging.

## Current descriptions of HIV-associated vasculopathy

## Aneurysmal disease

Since the first report of Salmonella-related mycotic aneurysmal disease by Du Pont *et al.*^24^ in an HIV-positive patient in 1989, increasing numbers of reports have emanated from Zimbabwe, Zambia and South Africa,^4^,^25^-^31^ confirming the occurrence of aneurysmal disease independent of bacterial infection. These aneurysms are multiple, with a predilection for young individuals and atypical locations, including the aorta, carotid, popliteal and femoral vessels.^28^,^29^,^31^ More recently, a predilection for femoral artery involvement has been documented.^4^ These aneurysms occur in the advanced stages of HIV disease, as demonstrated by low CD4 counts and systemic clinical features.^3^,^4^

## Clinical manifestations

**Fig. 2.**
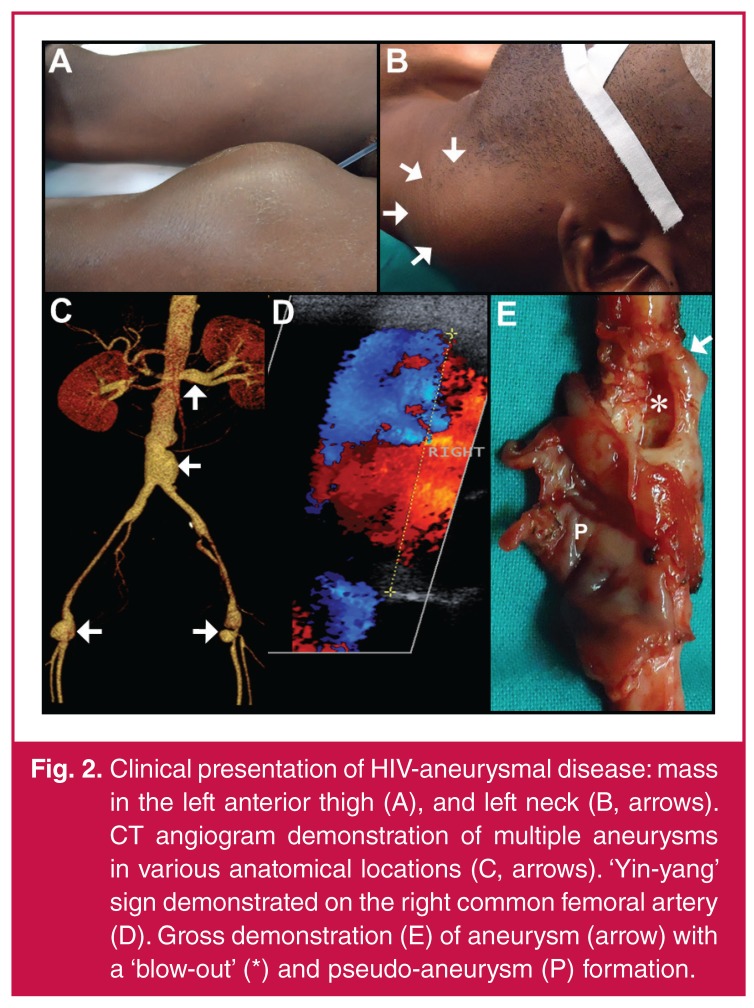
Clinical presentation of HIV-aneurysmal disease: mass in the left anterior thigh (A), and left neck (B, arrows). CT angiogram demonstration of multiple aneurysms in various anatomical locations (C, arrows). ‘Yin-yang’ sign demonstrated on the right common femoral artery (D). Gross demonstration (E) of aneurysm (arrow) with a ‘blow-out’ (*) and pseudo-aneurysm (P) formation.

Patients with aneurysms can be asymptomatic. However, when presenting as a space-occupying lesion, the clinical features are governed by the rate of growth, expansion and anatomical location. The presence of a pulsatile mass (Fig. 2A) may be complemented by constitutional findings of weight loss, associated lymphadenopathy and/or the presence of opportunistic infections. The symptom complex entails a varying spectrum, from pain in the majority of patients, to the associated effects of mechanical compression. An expanding carotid aneurysm (Fig. 2B) may result in dysphagia, stridor, hoarseness of voice, cranial nerve palsies, a hemispheric event as a consequence of thrombo-embolisation or frank aneurysmal rupture producing haemodynamic instability. Peripheral aneurysms may be associated with venous thrombosis as a result of venous compression by the aneurysmal mass.

## Imaging studies

Studies have shown aneurysmal transformation of the carotid, aortic, femoral and popliteal vessels.^3^,^4^ These aneurysms are multiple (usually more than three) (Fig. 2C) with a greater frequency in the carotid and femoral vessels. Doppler studies demonstrating the imaging features in HIV-associated vasculopathy are documented uncommonly. Woolgar *et al.*32 described the imaging characteristics with the aid of duplex ultrasound in HIV-related aneurysms in 12 patients. This modality has proven to be a valuable non-invasive screening tool.

As a general principle, aneurysms found clinically at one location guided the screening for aneurysms at other locations. Duplex ultrasound features characterised by forward and backward flow within a pseudo-aneurysm are reflected as a ‘yin-yang’ sign^32^ (Fig. 2D), cavitational echogenicity and turbulent flow with a vessel wall defect (Fig. 2E).^32^ This pathological process is eclipsed by adjacent hypo-echoic spotting and vessel wall thickening with normal proximal and distal vasculature. Patients deemed suitable for surgery are subjected to definitive imaging in the form of angiography or computerised tomographic angiography. Angiographically, the aneurysms are usually multiple, saccular or pseudo-aneurysmal with a variable location. The uninvolved arterial segments are pristine with a smooth vessel contour (Fig. 2C).

## Pathology

HIV-associated vasculopathy has a predilection for medium and large vessels. Its pathological profile has been compared to Takayasu’s disease because it affects young individuals and shares similar disease distribution and transmural vessel involvement. Histopathological vascular changes in AIDS were initially described by Joshi *et al.*^5^ in autopsies of children. Small and medium-sized vessels in six children demonstrated intimal fibrosis, elastic fragmentation, medial calcification, luminal narrowing and perivasculitis.^5^,^33^

By contrast, Calabrese *et al.*^34^ documented a systemic necrotising vasculitis following HIV infection in 11/14 patients with small-vessel involvement and lymphocytic infiltration. These features overlapped with polyarteritis nodosa, angiocentric lymphoproliferative disorder and primary angiitis of the nervous system.^34^ Marks and Kuskov^28^ demonstrated peri-arteritic fibroproliferative granulomatous inflammation of the aortic and iliac vessels in 5/12 patients with HIV-associated aneurysms, and hypothesised that most HIV patients develop a necrotising vasculitis of the vessel wall followed by the development of false aneurysms.

Some autopsy case studies and series of HIV-associated intracranial aneurysms,^35^-^45^ documented mainly in children, report similar microscopic features to those in extracranial vessels. While this observation suggests that intracranial aneurysmal pathology is a continuum of the same disease process, some workers, however, have noted differences as follows:

• variable absence of internal elastic lamina fragmentation
• medial thickening with sub-intimal SMC deposition
• controversial identification of viral protein, specifically gp 41, in the macrophages of the arterial wall, with inflammatory sparing of smaller leptomeningeal and parenchymal vessels
• absence of vasa vasora in the intracranial vessels, suggestive of a mechanism other than a leucocytoclastic vasculitis
• identification of specific vasculitic agents, such as varicella zoster virus, in lesional tissue.

**Fig. 3.**
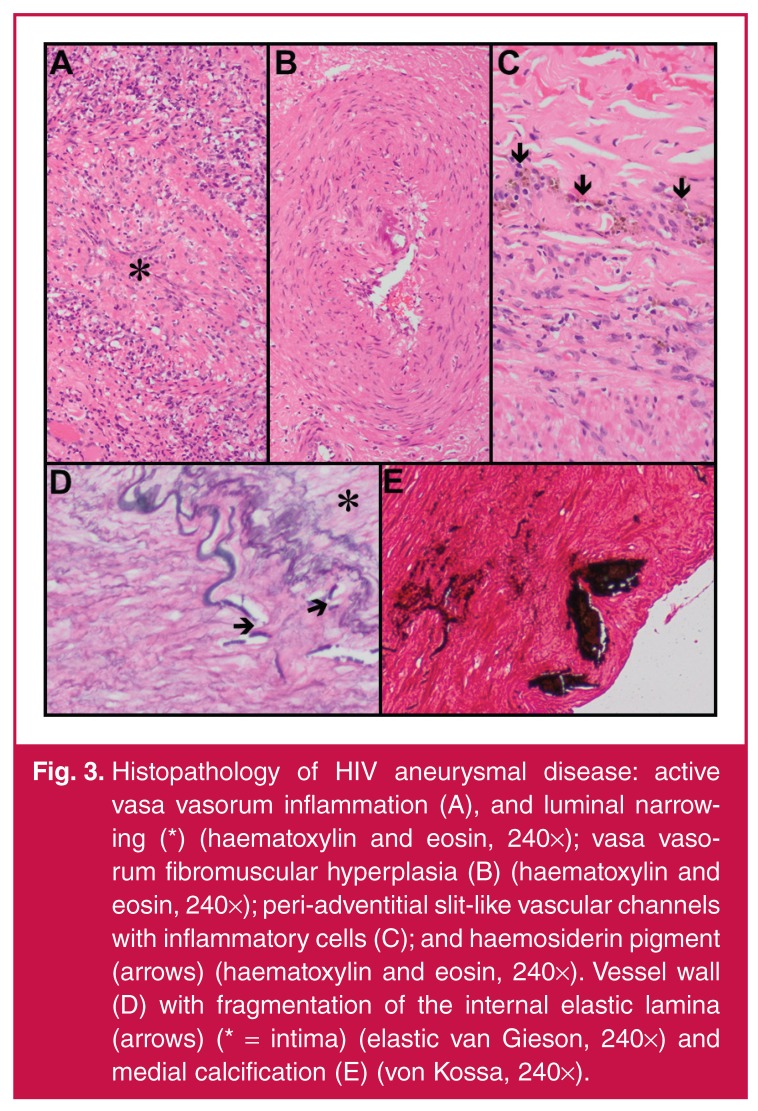
Histopathology of HIV aneurysmal disease: active vasa vasorum inflammation (A), and luminal narrowing (*) (haematoxylin and eosin, 240×); vasa vasorum fibromuscular hyperplasia (B) (haematoxylin and eosin, 240×); peri-adventitial slit-like vascular channels with inflammatory cells (C); and haemosiderin pigment (arrows) (haematoxylin and eosin, 240×). Vessel wall (D) with fragmentation of the internal elastic lamina (arrows) (* = intima) (elastic van Gieson, 240×) and medial calcification (E) (von Kossa, 240×).

In our unit Nair *et al.*^29^ studied the histopathological features of the aneurysm wall in detail in 10 patients. The common theme that was documented was inflammation of the vessel walls with the vasa vasora being the epicentre of this inflammatory process. Active lesions demonstrate inflammatory changes, necrosis and luminal narrowing (Fig. 3A), while inactive lesions are characterised by chronic features, including fibrosis and haemosiderin deposition. The media displays fragmented elastic fibres, variable loss of smooth muscle and fibromuscular hyperplasia (Fig. 3B). Intense involvement of the vasa vasora is hypothesised to cause transmural ischaemic necrosis. The adventitia demonstrates evolving inflammatory changes, characterised by macrophage infiltration with haemosiderin deposits.

Similarly, large-vessel vasculopathy has also been characterised by adventitial, medial and intimal alterations.^6^,^46^ Leucocytoclastic vasculitis of the vasa vasora and periadventitial vessels, proliferation of slit-like vascular channels, chronic inflammation and fibrosis are seen in the adventitia (Fig. 3C). While medial fibrosis, muscle damage, elastic fragmentation and intimal duplication are also present, the intima demonstrates fragmentation of the internal elastic lamina (Fig. 3D) and calcification (Fig. 3E).

Bacterial infection is also hypothesised to play a minor pathogenetic role. Although secondary infection caused by bacteria, viruses and fungi have been implicated, this has not been conclusively demonstrated. In the series by Nair *et al.*,^29^ micro-organisms were not identified, while in that of Marks and Kuskov,^28^ *Staphylococcus aureus* was isolated from peri-aneurysmal exudate. It is debatable whether the latter was a surface contaminant.

## Management

In the current era, HIV-infected patients presenting with vascular pathology are managed by the standard guidelines of HIV-naïve patients, with conservative management being reserved for patients with full-blown AIDS.^3^,^4^,^26^,^47^-^49^ The overall management of these patients poses a moral and ethical dilemma with regard to the appropriateness and timing of surgical intervention. At present there are no universal guidelines. Emergencies are prioritised irrespective of immune status. The majority of patients are young, fit and able to tolerate major surgery.

**Fig. 4.**
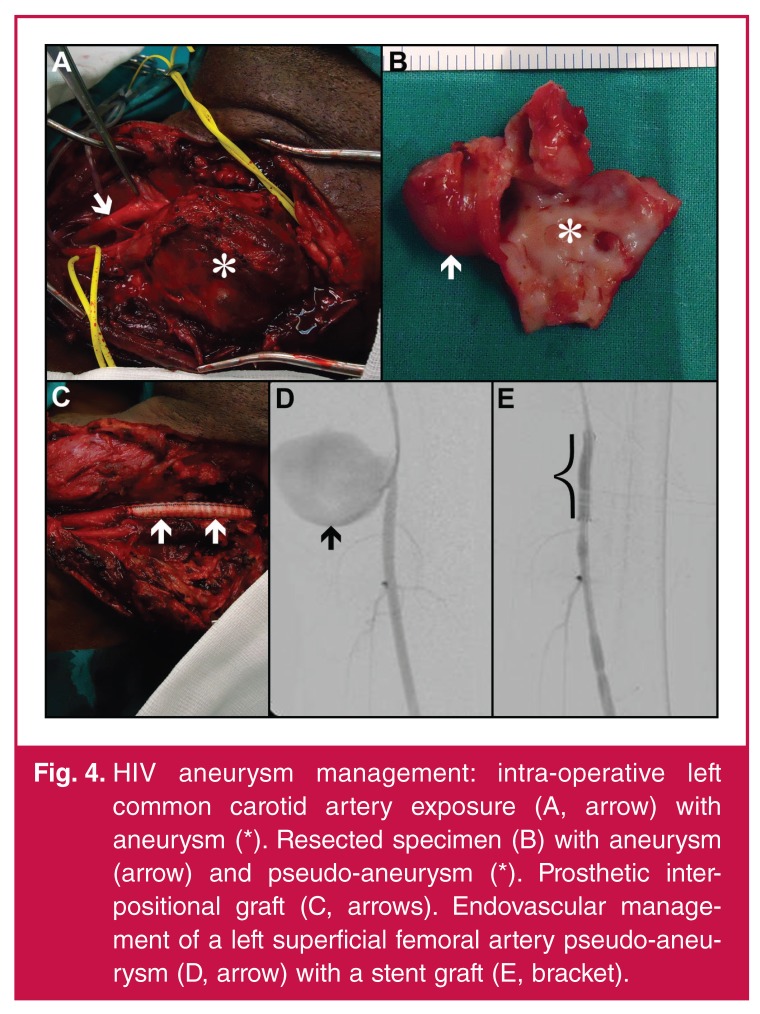
HIV aneurysm management: intra-operative left common carotid artery exposure (A, arrow) with aneurysm (*). Resected specimen (B) with aneurysm (arrow) and pseudo-aneurysm (*). Prosthetic interpositional graft (C, arrows). Endovascular management of a left superficial femoral artery pseudo-aneurysm (D, arrow) with a stent graft (E, bracket).

Treatment should be individualised and priority given to patients with symptomatic aneurysms. Intra-operatively, false or true aneurysms are identified (Fig. 4A).^3^,^4^,^26^ Intervention is offered for symptomatic aneurysmal lesions, and involves either ligation of vessels in septic lesions and occluded distal vessels, or resection (Figs 4B) and restoration of arterial continuity (Fig. 4c) following aneurysmal excision.^3^,^4^ The ligation of carotid lesions seems to be well tolerated, as evidenced by Nair *et al*.,^29^ with little or no neurological sequelae peri-operatively. The choice of conduit, namely prosthetic or autogenous grafts, for surgical bypass remains controversial.^29^ The latter is plagued by the associated risk of deep-vein thrombosis in these patients.

In selected patients with appropriate technical imaging criteria and poor physiological reserves, endovascular management with a stent-graft (Fig. 4D, E) constitutes a suitable alternative. Anecdotal reports with small patient numbers have documented its selected use and immediate success.^26^,^50^,^51^ Complications of this modality include stent-graft sepsis, occlusion, endoleaks and missed opportunistic infections.^4^,^52^ Scholtz^53^ has raised concerns about this modality, from a radiological perspective, with regard to angiographic access, small-calibre vessels and contrast pooling in the multiple aneurysms. Despite these reservations, it remains an attractive alternative because it promotes flexibility of treatment options. Endovascular intervention represents technology in evolution, with unknown long-term results. Patient selection is therefore crucial; the procedure should be reserved for patients with poor physiological reserves.

There have been no comparative studies, to date, on surgery versus endovascular intervention in patients with HIV vasculopathy. Expertise in this sphere is at present anecdotal and has been confined to isolated case reports.^54^,^55^ From a technical perspective, these aneurysms may present a challenge in relation to their large size, vessel tortuosity, flow dynamics and specific anatomical location, especially in relation to outflow tracts that may originate in close proximity to, or from the aneurysm sac itself. The branch vessels of interest in this instance would include the vertebral, internal iliac and visceral arteries. The rationale for endovascular intervention entails aneurysmal exclusion of the target vessel and preservation of laminar flow in the outflow tract. Endovascular devices to exclude these aneurysms include the use of covered or uncovered stents in the form of multi-layered compact cobalt,^55^ or open-cell nitinol mesh design. More recently, a novel idea of aneurysm exclusion using multi-layered stent technology without compromising branch vessel patency has been reported in HIV-infected patients. The basis for this technique has been extrapolated from the pipeline embolisation device,^54^ which is used to treat intracranial aneurysms. Its successful usage has been described by Euringer *et al.*^55^ in a patient with multiple HIV-related aneurysms. The deployment of this type of stent achieves aneurysm exclusion and restoration of laminar flow with aneurysm autothrombosis as a result of strangulated flow.

This technique can, in theory, also be augmented with coiling to ensure complete thrombosis within the aneurysm sac. Success when utilising the multi-layered stent in this setting requires definition because the aneurysmal sac can be completely excluded with total sac content thrombosis or sac shrinkage with sluggish flow. According to the authors,^55^,^56^ the merit in this technique facilitates continuous branch vessel patency, especially the patency of visceral branches of the aorta. Sustained patency may require the use of long-term antiplatelet therapeutic agents such as clopidogrel. The effect of this type of anti-platelet therapy may be negated by some anti-retroviral agents,^54^ but it is possible to identify patients with this form of resistance prior to intervention.

The multi-layered stent has the technical limitations of a compact strut design, which precludes additive coiling and potential excessive foreshortening during deployment. This may result in a geographical miss. Although the endovascular approach is cushioned and not plagued by the physiological challenges of robust anaesthesia, blood loss and the peri-operative complications of contamination, wound sepsis and transfusion requirements during operative surgery, the attractiveness is evidenced by a shorter hospital stay, and access to a lesion from a remote site free of contamination. The long-term durability of this form of intervention is unknown at present.

## Prognosis and outcome

The laboratory features are characterised by deranged CD4 counts, hyperglobulinaemia, inverted CD4/CD8 ratios and hypoalbuminaemia. 29,31,47 Van Marle *et al*.^47^ attempted to correlate some of these parameters to surgical outcome and revealed that these markers were associated with a poorer prognosis, while Robbs and Paruk^4^ failed to demonstrate a correlation.

Peri-operative mortality rates have ranged from 9–10.6% in South Africa^4^ to 33% in Houston,^57^ where the majority (56%) of patients were intravenous drug abusers with challenges related to wound healing and sepsis. Lin *et al.*^57^ reported a late graft sepsis rate of 10% with prosthethic graft usage.

The long-term results are largely unknown as follow-up remains a problem. Furthermore, the majority of the reported cases were conducted in the pre-HAART era.

## Occlusive disease

Occlusive disease is a less studied entity that shares microscopic and laboratory features with its aneurysmal counterpart. It has an affinity for young males under 40 years of age. The limbs are usually involved, with the lower limbs involved more frequently than the upper limbs.^3^,^4^,^47^,^48^,^58^-^60^ The classic risk factors for occlusive vascular disease are less prevalent.^46^,^58^

## Clinical manifestations

**Fig. 5.**
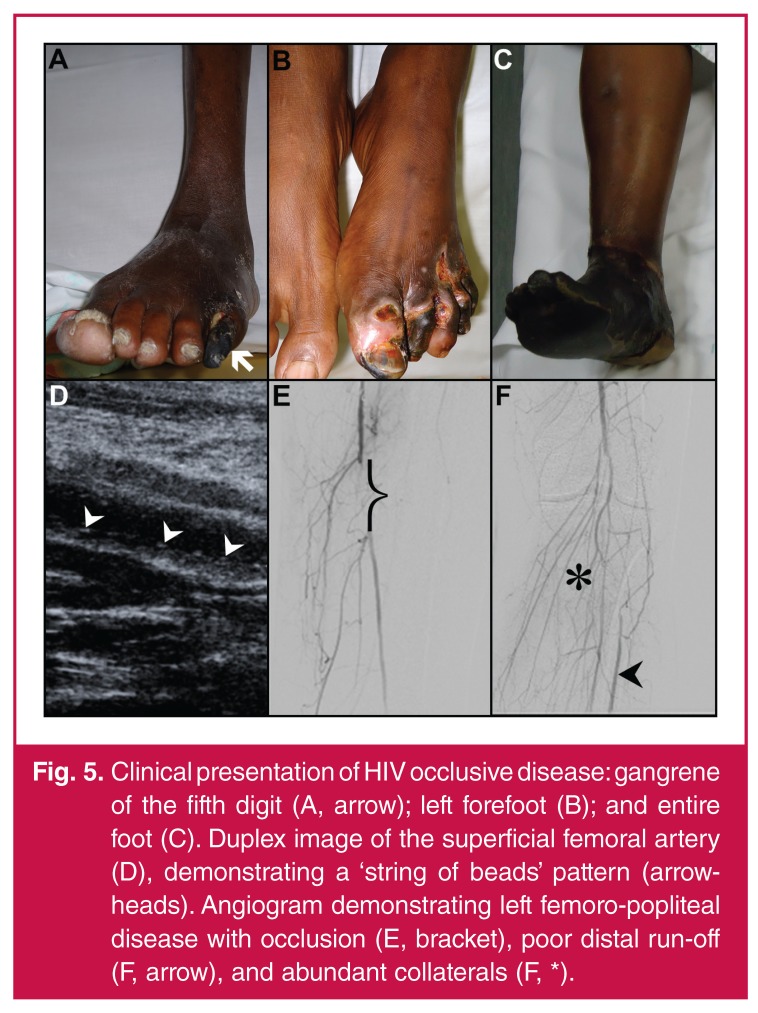
Clinical presentation of HIV occlusive disease: gangrene of the fifth digit (A, arrow); left forefoot (B); and entire foot (C). Duplex image of the superficial femoral artery (D), demonstrating a ‘string of beads’ pattern (arrowheads). Angiogram demonstrating left femoro-popliteal disease with occlusion (E, bracket), poor distal run-off (F, arrow), and abundant collaterals (F, *).

Patients may manifest acutely with primary thrombosis and clinical features of acute arterial occlusion. Chronic disease may present with features of critical ischaemia in the form of rest pain or gangrene in more than 50% of patients (Fig. 5A–C). Anatomically, infra-inguinal disease is more common than aorto-iliac disease.

## Imaging studies

Duplex studies have demonstrated typical linear sub-intimal deposition of calcium in the vessel wall, classically described as a ‘string of beads’^32^ appearance (Fig. 5D), together with evidence of intraluminal thrombus in patients presenting acutely. Mulaudzi *et al.*^22^ documented that additional imaging in this group of patients was non-contributory.

Invasive imaging using computed tomographic studies and angiography have shown that the contralateral vessels are usually disease free while the symptomatic limb vessels demonstrate multi-segment involvement, long-segment occlusions (Fig. 5E), poor distal run-off and an abundance of well-established collaterals (Fig. 5F).^3^,^4^,^47^,^48^,^58^,^59^

## Pathology

**Fig. 6.**
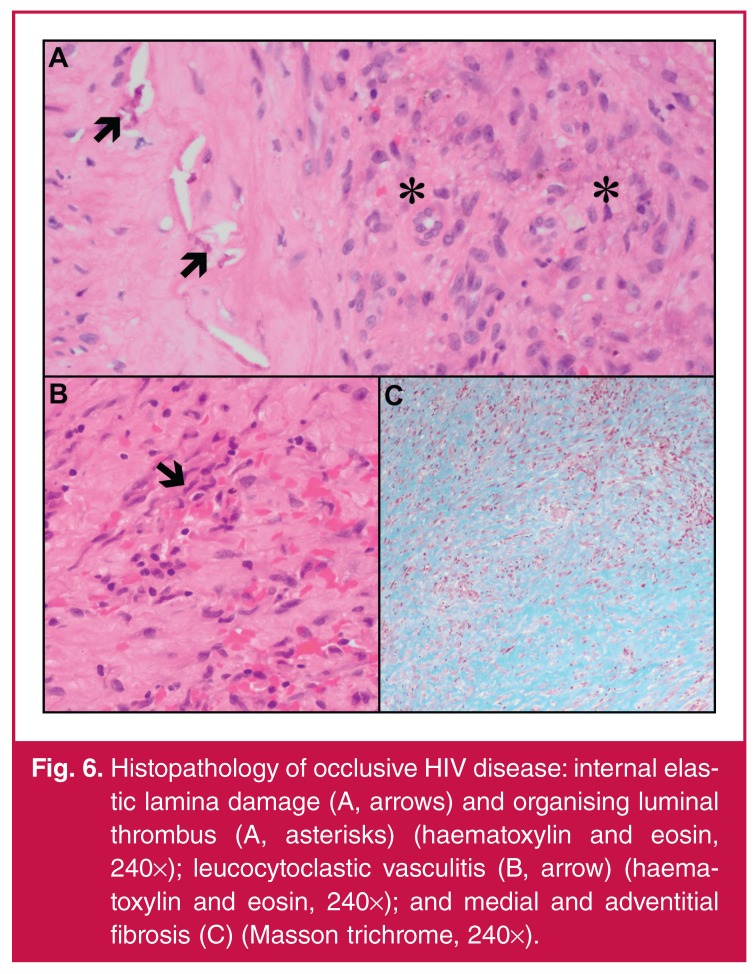
Histopathology of occlusive HIV disease: internal elastic lamina damage (A, arrows) and organising luminal thrombus (A, asterisks) (haematoxylin and eosin, 240×); leucocytoclastic vasculitis (B, arrow) (haematoxylin and eosin, 240×); and medial and adventitial fibrosis (C) (Masson trichrome, 240×).

In the series by Mulaudzi *et al.*,^22^ 36% of patients (*n* = 8) who had histopathological investigations had organised bland thrombus and an intense inflammatory reaction in the vessel lumen. On microscopic analysis of the occlusive lesions, medial scattered chronic inflammatory cells, focal medial calcification, destruction of the internal elastic lamina (Fig. 6A) and medial muscle, leucocytoclastic vasculitis of the vasa vasora (Fig. 6B), mural fibrosis (Fig. 6C) and luminal organising thrombus (Fig. 6A) have been noted. In addition, viral proteins on the lymphocytes of arterial and aneurysmal tissue were seen but atherosclerosis was not identified.

Nair *et al.*^59^ found no evidence of atherosclerotic involvement of the vessel wall during macroscopic examination at surgery. Autopsy studies performed by Micheletti *et al.*^61^ on donor coronary vessels of 10 HIV-positive patients revealed linear calcium deposition in the internal elastic lamina, independent of intimal atherosclerosis and calcification, a microscopic feature supposedly unique to HIV-infected individuals. This feature is theorised to reflect arterial stiffening and may be associated with premature vascular aging and chronic illness in HIV-infected patients.

## Management

The management of HIV-infected patients who present with vascular pathology is congruent with the standard guidelines of HIV-naïve patients, with conservative management being reserved for patients with full-blown AIDS.^3^,^4^,^47^,^48^,^58^ Those patients presenting with acute arterial occlusion as a result of primary thrombosis are characterised by unfavourable outcomes with embolectomy. This is borne out in the study by Mulaudzi *et al.*,^22^ who demonstrated that embolectomy was often followed by re-thrombosis within 48 hours. In his study, 17/22 patients were treated by ablation, with a limb salvage rate of 27%.^22^ A possible reason for the poor outcome was explained by the persistence of the underlying vasculitic process despite management of the obstructing lesion.

Patients with chronic disease are imaged and treated with surgical bypass, catheter-directed therapy, or an ablation for unsalvageable limbs. Post-operative wound healing and graft sepsis is not unusual.^48^ To overcome this shortcoming, van Marle *et al.*^58^ used silver-impregnated grafts for surgical bypass. Immediate post-operative results were favourable.

## Prognosis and outcome

Attempts have been made to correlate serum albumin and CD4 counts with postoperative outcome in these patients but the results vary. Although a low CD4 count in association with hypoalbuminaemia correlated with a poor postoperative outcome,^58^ the overall 30-day mortality rate for acute and chronic occlusive disease attained by Robbs and Paruk^4^ was 23%, compared to a long-term mortality of 28.75% by van Marle *et al.*^58^ Furthermore, improved long-term survival in this grouping was negated by poor limb-salvage rates of 36.1%, with poor distal run-off being a contributory factor that precluded surgical bypass.^58^

## Other HIV-associated vascular manifestations

## Spontaneous arteriovenous fistulae

Arteriovenous fistulae may occur as a result of trauma or endovascular procedures. Spontaneous arteriovenous fistulae following HIV infection are rare, with anecdotal experiences reported in the literature.^48^,^62^ A case report detailing this clinical scenario related to a young patient presenting with a pulsatile mass of his right lower thigh.^62^ Angiography revealed a distal superficial artery lesion with pooling of contrast and delayed venous filling. The patient was treated surgically, with a successful outcome. Microscopy of the arterial wall with regard to the index patient demonstrated features similar to that observed in aneurysmal and occlusive disease.

## Spontaneous cervical artery dissection

An isolated case report described a spontaneous cervical artery dissection.^63^ This pathology was observed in the vertebral artery. The speculated pathogenesis was a structural defect in the arterial wall. Deficiencies of micronutrients, folate and cobalamine have been observed in HIV-infected patients.^63^ These deficiencies result in high circulating homocysteine levels that are thought to adversely affect the elastin content of the vessel wall, rendering it potentially vulnerable to a dissection.

## Atherosclerosis in HIV-infected patients

Evidence demonstrates that endothelial injury in HIV-infected patients occurs as a result of progression and severity of HIV infection *per se*.^14^ However, more recently, atherosclerosis has been documented following HIV infection and its management with HAART.^64^-^70^

## The relationship between atherosclerosis and HIV infection

Atherosclerotic disease is essentially an inflammatory event in the setting of classic cardiovascular risk factors, namely, smoking, hyperlipidaemia, family history, diabetes and hypertension. Accelerated atherosclerosis may evolve from the metabolic changes accompanying HIV infection, inclusive of hypercholesterolaemia, decreased high-density lipoprotein (HDL) cholesterol, elevated C-reactive protein levels and increased fibrinogen and plasminogen-activating inhibitor activity. Patients with these metabolic changes are more prone to coronary artery disease.^64^,^65^ In addition, cigarette smoking is a contributory factor.^70^

**Fig. 7.**
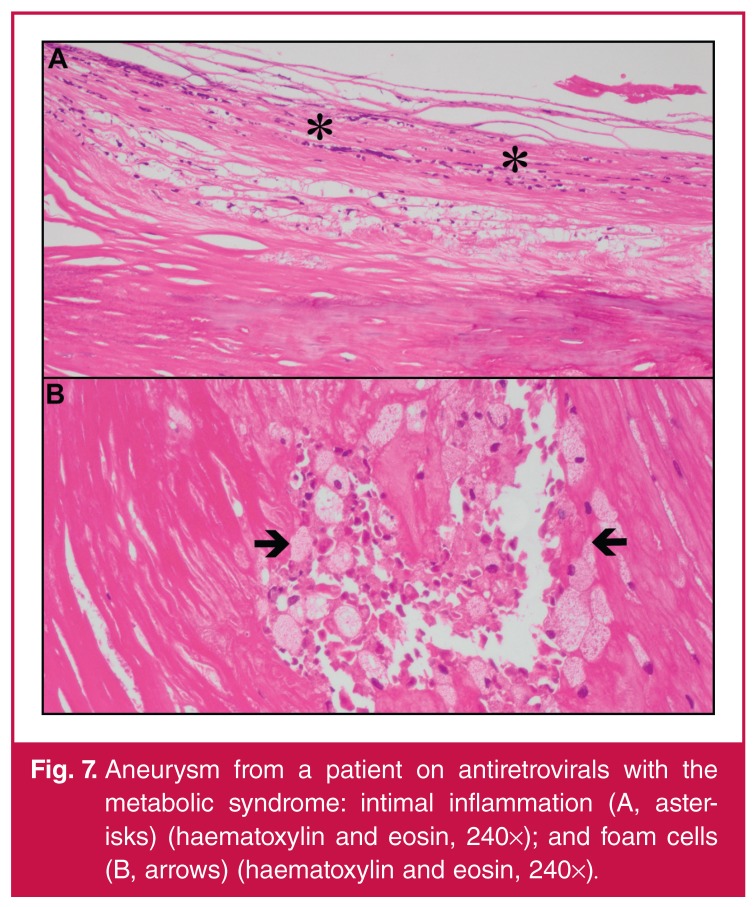
Aneurysm from a patient on antiretrovirals with the metabolic syndrome: intimal inflammation (A, asterisks) (haematoxylin and eosin, 240×); and foam cells (B, arrows) (haematoxylin and eosin, 240×).

It has been hypothesised that atherosclerotic disease in HIV-infected patients is divided into two distinct phases.69 The first includes vessel wall inflammation (Fig. 7A, B), which cascades towards classic atheromatous features. The second includes progression of these morphological changes that are sustained by classic atheromatous risk factors.

## The relationship between atherosclerosis and HAART

In the current era of effective HAART, cardiovascular risk has emerged as a significant marker of morbidity and mortality among surviving HIV-positive patients. It is questionable whether HIV itself confers a significant cardiovascular risk, as some studies show conflicting results.^68^ Surrogate markers such as carotid intimo-medial thickness and endothelial dysfunction implicate HIV as an independent variable for cardiovascular risk. The potential mechanisms for coronary artery disease in HIV-infected patients arise from viral proteins that attract monocytes to the vessel wall, activation of which stimulates inflammation and retardation of cholesterol efflux.^68^

HAART acts through enzymatic inhibition of HIV. The currently available agents used to treat HIV-infected patients are classified into protease inhibitors, nucleoside and non-nucleoside analogues.^64^ The protease inhibitors cause metabolic complications, including hyperlipidaemia, central fat accumulation and insulin resistance, while the nucleoside inhibitors cause lipo-atrophy and mitochondrial damage. Non-nucleoside analogues cause lipid elevations. These complications are associated with a 26% risk of myocardial infarction per year on combination therapy.^66^ Newer agents without the risk of lipid elevation, are presently being studied. It remains to be seen whether this translates into cardiovascular risk reduction.

It is debatable, however, whether HAART contributes to endothelial injury. Studies supportive of the concept propose a triad composed of the virus, immune reconstitution and HAART, which initiates premature endothelial activation. This collaborative influence, which may affect the structural composition of arterial lesions in HIV patients, is borne out by variation in structural atheromatous changes as opposed to classical atheroma. This includes the presence of vessel wall inflammation, ultrasonographic variations and impaired flow-mediated dilatation.^68^ The latter reflects an inverse relation between the viral load and endothelium-mediated vasodilation in association with some HAART agents. This produces altered ankle–brachial indices (ABIs) that are regulated by the magnitude of dyslipidaemia.

The spectrum of atherogenic exposure spans short-term exposure reflected in acute impairment of brachial artery flow-mediated dilatation, and long-term intimo-medial thickness in the carotid vasculature.^67^,^71^ These features are reflective of a metabolic atheromatous process that is governed by the HIV load.^69^

The availability of HAART has dampened the effect of opportunistic infections and promoted longevity in HIV-infected patients. As a result, HIV-infected patients have become increasingly prone to cardiovascular manifestations, particularly premature atherosclerosis, which is not associated with the conventional predisposing risk factors that are emphasised in HIV-naïve patients.^64^,^65^ HIV vasculopathy usually presents in the advanced stages of the disease.^65^,^72^

Robust data on the cardiovascular implications of viral infection is largely unknown, as most research has focused on Caucasians infected with the HIV-B sub-type virus. The Hypertension in Africa Research Team^73^ conducted a prospective epidemiological study in 300 urban and rural patients and control subjects. In this study, the preliminary findings in untreated subjects demonstrated endothelial injury following viral invasion that resulted in endothelial inflammation and dysfunction, with elevated molecular markers and accelerated atherosclerosis that was exacerbated by low levels of HDL cholesterol. In the older patients there was evidence of rapid vascular aging, suggestive of diminishing vascular function as determined by pulse-wave velocity studies.

The treatment group on HAART demonstrated elevated systolic blood pressure without established systemic hypertension and stabilised lipid profiles with lipodystrophic changes. Soluble urokinase plasminogen activator, a soluble protein biomarker of progressive inflammation in HIV-1 infected patients, is a key mediator between inflammatory and metabolic derangements. The levels were significantly elevated in treated, compared to untreated and control African patients, signifying a correlation with lipodystrophic changes following HAART over the three-year follow-up study. The study results seem to suggest a deteriorating profile irrespective of HAART in the South African black population. Therefore greater insight into the adverse cardiovascular influence on the South African HIV population is necessary.^73^,^74^

Coronary artery calcium scoring, a non-invasive surrogate marker of atherosclerosis, serves as a predictor of myocardial infarction and coronary mortality. Studies in this sphere in HIV-infected patients are lacking. One reported study demonstrated a 6–8% incidence of coronary calcification. Although HIV patients on HAART had higher calcium scores, these levels were, in effect, lower than those in untreated patients.^67^

A clinical manifestation of premature atherosclerosis is peripheral vascular disease (PVD). This is an emerging HIV-associated disorder with an unknown prevalence that has assumed prominence following the widespread availability of HAART. In a Swiss pilot study of 92 HIV-infected patients, Periard *et al.*^75^ detected a 20% (*n* = 19) incidence of subclinical atherosclerosis. Although these patients had normal resting ABIs, the exercise ankle systolic pressure (ASP) and ABI were deranged. The possible mechanisms underlying PVD relates to lifestyle-induced cardiovascular risk factors, combinations of antiretroviral therapeutic agents and HIV per se causing inflammatory lesions.

## Management

The medical management of HIV-associated vasculopathy includes HAART, control of hyperlipidaemia and eradication of traditional risk factors.

• The International AIDS society of USA recommends the use of HAART in asymptomatic individuals with CD4 cell counts < 350 cells/mm^3^. Other indications include a high viral load of > 100 000 copies/ml, active hepatitis B or C infections, and evidence of HIV nephropathy.^67^,^76^ The main objective of the initial choice of regime relates to viral suppression but adverse effects of the drug profiles should be considered in patients at high cardiovascular risk.
• Current recommendations, including lifestyle modifications such as dietary and exercise interventions, have demonstrated decreased lipid values by 11–25% in HIV-infected patients. It is yet to be determined whether hyperlipidaemia in HIV-infected subjects should be considered a separate cardiovascular risk factor. In patients with carotid stenosis, statins have reduced intimo-medial thickness and cerebrovascular events, and have beneficial anti-inflammatory and pleiotropic properties.^77^ It is unknown whether these effects will materialise in HIV-infected patients beyond its lipid-lowering potential.
• The classic risk factors, including smoking, diabetes, hypertension and hyperlipidaemia, are assuming greater significance in the HIV population.^78^ The atherosclerotic burden may be worsened under these circumstances, particularly in association with HAART. Patients should be counselled to stop smoking. Psychological and medicinal measures should be instituted, if indicated, together with medical optimisation of diabetes and hypertension, as in HIV-naïve patients.

The SMART study^79^ has shown that the risks outweigh the benefits in subjects on prolonged HAART who have elevated inflammatory markers. In addition, low CD4 counts were associated with increased surrogate markers for atherosclerosis and cardiovascular complications. Despite viral suppression, residual immunological effects may still confer a cardiovascular risk, which, in part, may be related to gut bacterial translocation.^79^ Current therapeutic options that are being explored to negate this adverse influence include novel therapies to modify T-cell activation and senescence, immunomodulation, and nutritional supplements to restore the gut flora. Although it is thought that short-term HAART may reduce cardiovascular risk, it is not known whether it will completely reverse HIV-related cardiovascular disease in the long term.

## Current challenges

The literature pertaining to the diverse spectrum of HIV-associated large-vessel vasculopathy has been confined to case reports,^21^,^24^,^50^,^62^,^63^ small patient series,^26^,^30^ and larger studies^4^,^22^,^28^,^29^,^31^,^47^,^52^,^57^-^59^ (Table 1). The majority of caseloads have focused on clinical aspects of aneurysmal and occlusive disease that were observed mainly during the pre-HAART era, while, to date, the mechanisms underlying the pathological process remain theoretical without definitive isolation of an infective agent. Limitations in terms of patient numbers, inconsistencies in respect of specimen sampling for hypercoagulation, thrombophilic screens, histopathological sampling and microbiological and viral analyses have resulted in an incomplete understanding of HIV-associated vascular disease.

**Table 1:** Literature review of HIV-associated vasculopathy series^4^,^22^,^28^-^31^,^47^-^48^,^52^,^57^-^59^

		*Aneurysms*		*Occlusive disease*	
	*Publication year*	*n*	*MR (%)*	*n*	*MR (%)*
Marks and Kushov^28^	1995	12	ND	0	N/A
Nair, *et al.*^29^	1999	10	30	0	N/A
Nair, *et al.*^30^	2000	4	25	0	N/A
Nair, *et al.*^30^	2000	0	N/A	22	0
Nair, *et al.*^30^	2000	28	7	0	N/A
Van Marle, *et al.*^47^	2002	9	0	24	0
Lin, *et al.*^57^	2004	20	33	28	15
Mulaudzi, *et al.*^22^	2005	0	N/A	22	14
Botes and van Marle^48^	2007	24	10.6	66	3.6
Van Marle, *et al.*^58^	2009	0	N/A	91	20
Robbs and Paruk^4^	2010	111	9	115	11
Padayachy and Robbs^52^	2012	22	14	0	N/A

MR: mortality rate; *n*: patient numbers; N/A: not applicable; ND: not documented, %: patient percentage.

In future studies, attempts should be made to obtain representative diseased and juxtaposed, apparently normal, arterial wall samples to facilitate an improved understanding of the established and evolving disease spectrum, respectively. Furthermore, molecular microbiological techniques for isolation and identification of infective organisms need ongoing development and optimisation to keep abreast with emerging technology.

## Future directions

The vascular endothelium and smooth muscle cell components are potential keys to unravelling some of the mysteries relating to the pathophysiology and microscopic changes in HIV-associated vasculopathy. Animal models such as the transgenic mice model have been used to simulate arterial wall pathology following HIV invasion. This seems promising for improved understanding of the possible disease pathogenesis.

Various molecular markers and receptors are being studied at a cellular level with the aim of blocking the destructive biochemical pathways.^80^ Furthermore the availability of HAART, while offering a therapeutic solution, is not without its metabolic adversities that fuel the atherogenic process, thereby compounding risks. Newer agents with or without minimal metabolic complications are being investigated.

Presently there are no universal surgical guidelines for HIV-associated large-vessel vasculopathy. Surgical intervention remains the mainstay of therapy for aneurysmal and occlusive disease, however, with endovascular intervention reserved for a subset of patients with poor physiological reserves or who are too ill to tolerate anaesthesia. The exact role of endovascular intervention for this profile of patients requires definition and as yet there are no studies comparing surgery and endovascular intervention in HIV-infected patients. The prevalence of peripheral arterial disease in the HIV-positive population needs further investigation as asymptomatic patients manifest with subclinical atherosclerosis, as determined by deranged ABIs following exercise.^81^

## Conclusion

HIV is a pandemic with a widespread disease profile. The availability of HAART has offered a therapeutic lifeline for longevity but is negated by potential vascular complications. As a result the incidence of vasculopathic manifestations over the last decade has increased. The challenge lies in unravelling the elusive aetiological and histopathological mysteries that surround HIV-associated large-vessel vasculopathy, and in so doing, assist with improved insight and knowledge in devising potential future therapeutic modalities that will enable improved vascular surgical practice in this context.

## Key messages

• HIV infection is a multisystem disease with a diverse clinical spectrum.
• HIV-associated vasculopathy is a unique entity confined mainly to young individuals.
• The pathogenesis remains largely theoretical and entails a complex interplay between inflammatory, opportunistic infections and atherosclerotic components.
• The key histopathological findings include leucocytoclastic vasculitis of the vasa vasora, fragmentation of the internal elastic lamina, variable intimo-medial calcification, fibromuscular hyperplasia and transmural inflammation.
• The availability of HAART has altered the natural history of the disease profile, with atherosclerosis emerging as a potentially ominous therapeutic challenge.
• Patients are managed along standard vascular surgical guidelines as universal management guidelines have not reached consensus to date.
• Long-term results of intervention are uncertain because of suboptimal patient compliance.
• The exact indications for endovascular intervention require further study.
